# Fentanyl supplement expedites the onset time of sensory and motor blocking in interscalene lidocaine anesthesia

**Published:** 2010

**Authors:** RS. Moharari, J. Sadeghi, MR. Khajavi, ME. Davari, M. Mojtahedzadeh

**Affiliations:** 1 Department of Anesthesiology Faculty of Medicine Tehran University of Medical Sciences; 2 Shahid Moayeri Hospital Social Security Organization; 3 Department of Clinical Pharmacy Faculty of Pharmacy, Tehran University of Medical Science, Tehran, Iran

**Keywords:** Interscalene block, Regional anesthesia, Peripheral opioids effect

## Abstract

**Background and the purpose of the study:**

Opioids are usually used in regional anesthesia, with or without local anesthetics to improve the regional block or postoperative pain control. Since no data are available on fentanyl's effect on the onset time of lidocaine interscalene anesthesia, the purpose of this study was to examine its effect on the onset time of sensory and motor blockade during interscalene anesthesia.

**Methods:**

In a prospective, randomized, double-blind study, ninety patients scheduled for elective shoulder, arm and forearm surgeries under an interscalene brachial plexus block.They were randomly allocated to receive either 30 ml of 1.5% lidocaine with 1.5 ml of isotonic saline (control group, n=39) or 30 ml of 1.5% lidocaine with 1.5 ml (75 µg) of fentanyl (fentanyl group, n=41). Then the onset time of sensory and motor blockades of the shoulder, arm and forearm were evaluated every 60 sec. The onset time of the sensory and motor blockades was defined as the time between the last injection and the total abolition of the pinprick response and complete paralysis. The duration of sensory blocks were considered as the time interval between the administration of the local anesthetic and the first postoperative pain sensation.

**Results:**

Ten patients were excluded because of unsuccessful blockade or unbearable pain during the surgery. The onset time of the sensory block was significantly faster in the fentanyl group (186.54±62.71sec) compared with the control group (289.51±81.22, *P<*0.01). The onset times of the motor block up to complete paralysis in forearm flexion was significantly faster in the fentanyl group (260.61±119.91sec) than the control group (367.08±162.43sec, *P<*0.01). There was no difference in the duration of the sensory block between two groups.

**Conclusion:**

Results of the study showed that the combination of 75 µg fentanyl and 1.5% lidocaine solution accelerated the onset of sensory and motor blockade during interscalene anesthesia.

## INTRODUCTION

One of the major setbacks for brachial plexus blockade in various sites is the delayed onset time of neural blockade. Various methods, with limited success, have been adopted to reduce the delayed onset time of the neural blockade (1). Co-administration of opioids and local anesthetic solutions have been employed in order to expedite the onset time, improve the quality of blockages and prolong the duration of the peripheral nerve blockade (2, 3). However in some studies, it has been shown that opioids could not improve onset time of sensory blockade in supraclavicular and axillary block (4–7). Interscalene technique of brachial plexus block seems to be an unique model to evaluate combination of opioids and local anesthetics, because the drug is deposited at the roots. There are limited data indicating the fentanyl's impact on the onset time of lidocaine interscalene anesthesia. The purpose of this placebo- controlled clinical trial study was to examine the effect of fentanyl on the onset time of sensory and motor blockades during interscalene anesthesia.

## MATERIAL AND METHODS

This study was carried out in the framework of a prospective double blind clinical study on 90 ASA I-II patients’ who underwent surgical operations on their upper extremities. The age range of patients enrolled in this study were 18-60 years. Also, the study was approved by the committee of institutional ethics and a written informed consent was obtained from each patient before performing the interscalene blocking and surgery. The study exclusion criteria were set out as follow:

Patient's refusal, sensitivity to the local anesthetics, consuming anticoagulating drugs and the emergence of skin infection at needle insertion points, Body Mass Index exceeding 40 kg/m2, pre-existing neurological deficits, severe obstructive pulmonary disease, persisting acute pain and the existence of implanted electrical devices such as pacemakers and receiving any preoperative opioids or sedative drugs. Patients requiring high doses of opioids and general anesthesia during the surgery were excluded from the study.

Patients included in this study were randomly assigned via the sealed envelope method, to receive interscalene brachial plexus blockade using 30 ml of either 1.5% lidocaine plus 75 µg of fentanyl (fentanyl group) or the same dose of lidocaine plus 1.5 ml of the normal saline (control group). Drugs were prepared in identical syringes by a nurse who was not involved in caring or monitoring the patients and sealed in sequentially numbered, opaque envelopes that were opened before anesthesia. The patients, the attending anesthesiologists as well as the physicians and nurses of the recovery room, all remained blind to the drug assignments.

The blockings were created by one anesthesiologist via nerve stimulation using a 50 mm insulated needle and a stimulator (Stimuplex®; B Braun, Melsungen, Germany).

Prior to the needle insertion, all patients had their IV's secured. Routine monitoring of patients, including non-invasive arterial blood pressure, electrocardiogram and oxygen saturation, were conducted. The patients were placed in supine position while the arm to be anesthetized remained adducted and the head turned away toward the opposite side which had to be blocked. Upon aseptic skin preparation, the interscalene groove was identified relying on the landmarks described by Winnie (8) followed by local skin infiltration using 2 ml of 1% lidocaine and inserting the Stimuplex® needle into the interscalene groove at cricoid cartilage level (C6). The needle was directed smoothly in medial, caudal, and dorsal directions inside the brachial plexus sheath at roots of the plexus level.

The intensity of the stimulating current was initially set to deliver 2 mA with the duration of impulse 0.1 ms which was gradually decreased until an appropriate motor response was localized in a nerve radiating the area on which surgery was planned to be performed with a current of 0.2-0.6 mA. Then the local anesthetic solution was administered over a period of 1 min. An identical injection technique was applied to all patients.

Time zero (t=0) was defined as the time of removal of the insulated needle from the skin. The sensory block was assessed by the pinprick method at 60 sec intervals. Pinprick sensation was examined using a blunt 21-gauge needle in a proximal to distal way along the skin dermatomes C4-C7.

Pinprick response was assessed by one anesthesiologist on a 2-point scale (sensation/ numbness) and compared with the sensation felt on the opposite limb (9).

The motor function at proximal of the limb was tested by asking patients to abduct the arm at the shoulder joint against gravity (C5-C6), flexion (C5- C6) of the forearm at the elbow. The motor block at distal of the limb was evaluated by wrist flexion (C6-C7).

Assessment of motor block was derived from the technique described by Bromage on a three-point scale from 3 (normal muscular force), to 2 (movement against gravity) and 1 (complete paralysis) (9).

The onset time for sensory blockade was defined as the time between the end of injection and total disappearance of the pinprick response from C5 to C7 at forearm. Similarly, the onset time for the motor blockade was considered as the total paralysis of the forearm flexion and extension. The duration of the sensory blockade was the time interval between administration of the local anesthetic and appearance of the first postoperative pain. The operating surgeon assessed the operative site in terms of pain sensation using a surgical forceps. In the case that the patients reported the sensation of pain at this time, the blockade was described as unsuccessful and general anesthesia was induced by the attending anesthesiologist. When the blockade was successful, surgery proceeded as usual. Before interscalene block, none of the patients received premedication but during the surgery, all patients were sedated by administration of 10 mg of diazepam.

The primary pilot study with 10 patients receiving lidaocaine was performed to estimate the standard deviation of the outcome measure. The mean and standard deviation of our main outcome measure i.e. sensory block in the forearm were 221.3 and 64 sec respectively. A sample size of 32 was calculated for each group at the significance level of 0.05 and a power of 80% to detect a 60 sec difference in the onset of the sensory block in the forearm. It was finally decided to recruit 45 cases in each intervention group.

At the end of study and after gathering the data, the statistical analyses were performed with SPSS for Windows version 11.5. Data are presented as mean (95% confidence interval), and number (%). For comparison the mean onset time of sensory and motor complete block and demographic variables, data were analyzed by independent student's t- test. Chi square test was used for categorical variables. A p value<0.05 was considered statistically significant.

## RESULTS

Of 90 patients enrolled in the study, 10 were excluded from the evaluation. Four patients in both groups failed to achieve the surgical block, and 2 patients in fentanyl and 4 in the placebo group reported severe pain on incision and were given general anesthesia. There were no significant differences between the groups considering successful and failed anesthesia, and the overall block success rate was 89%.

**Figure 1:**
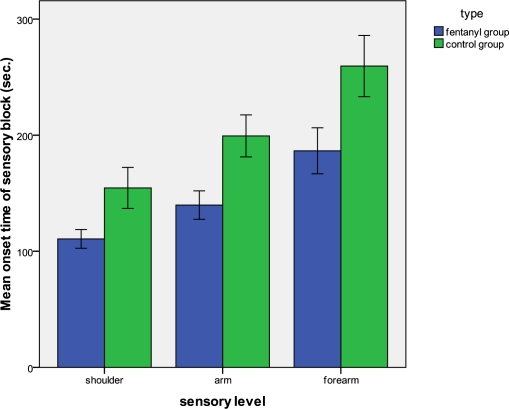
Mean onest time of sensory block in two groups.

**Figure 2:**
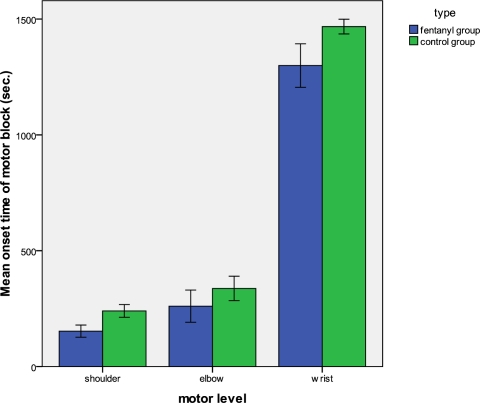
Mean onest time of motor block in two groups.

There were no differences in age, weight, height, sex, and the duration of surgery between the two groups (Table 1). The time to the onset of sensory and motor blockade for each group are shown in Table 2 (Figs. 1 and 2).

**Table 1:** Characteristic of Patients and Surgery.

	Fentanyl group (n=41) Mean±SD	Control group (n=39) Mean±SD	p value
Age (yr)	44.89±11.38	41.46±10.93	0.26
Weight (kg)	72.10±9.58	72.95±7.91	0.68
Height (cm)	168±7	176±6	0.61
*Sex* (M/F)	22/19	22/11	
**Type of surgery**			
ORIF humerus and elbow	14	12	0.18
Rotator cuff repair	17	14	0.23
Distal clavicle fixation	10	13	0.16
Duration of surgery (min)	95±18	91±16	0.24

**Table 2:** Onset time of sensory and motor blockade in each group.

Characters	Type of intervention				

	Lidocain+fentanyl Mean±SD	Lidocain Mean±SD	p Value*	Mean Difference	95% CI** of Mean Difference±SD
Sensory block in shoulder (sec)	110.63±25.54	154.59±54.62	0.01	−43.95±9.45	−62.78 to25.12
Sensory block in arm (sec)	139.80±38.82	199.38±55.72	0.01	−59.57±10.69	−80.86to−38.29
Sensory block in forearm (sec)	186.54±62.71	289.51±81.22	0.01	−72.97±16.17	−105.18to40.76
Onset of motor block in arm (sec)***	105.32±57.16	173.56±39.33	0.01	−68.24±11.02	−90.19to−46.29
Complete motor block in arm (sec)	152.90±83.40	240.08±84.94	0.01	−87.17±18.82	−124.65to−49.69
Onset of motor block in forearm flexion (sec)***	176.73±104.17	268.05±129.49	0.01	−91.31±26.21	−143.50to−39.13
Complete motor block in forearm flexion (sec)	260.61±219.91	337.08±162.43	0.82	−76.46 ±	−162.87to−9.94
First time of pain feeling (min)	121.10±7.62	119.92±5.52	0.43	−1.17±49.86	−1.80 to 4.14
Time of first analgesic Intervention (min)	142.22±7.38	137.67±6.35	0.004	−4.55±50.01	1.48 to 7.62

* Independent t-test

** Confidence Interval

*** Bromage Scale

The onset time of the sensory block was significantly faster in the fentanyl group (186.54±62.71 sec) than the control group (289.51±81.22 sec, P<0.01).

The onset times of the motor block up to complete paralysis in forearm flexion was significantly faster in the fentanyl group (260.61±119.91 sec) than in the control group (367.08±162.43 sec, P<0.01).The return time of pain feeling was not different between the two groups but the time that the patients asked for an analgesic was longer in patients who took fentanyl.

## DISCUSSION

This study demonstrates that addition of 75 µg of fentanyl to 1.5% lidocaine solution in an interscalene brachial plexus blockade accelerates the onset time of sensory and motor blockades.

According to Table 2, the complete motor block in the distal section (wrist) took a long time and since the surgery in this study was performed in the proximal part, it was started after a complete sensory and motor blockade of the forearm.

The pain could be effectively controlled by the central and peripheral actions of the opioids. Opioids bind to receptors on dorsal root ganglia, the central terminals of the primary afferent neurons and the peripheral sensory nerve fibers and their terminals. The structure of these receptors is very similar to their brain counterparts (10).

Regarding the possible mechanisms for acceleration, sensory and motor blockades created by fentanyl in this study, it is suggested that fentanyl might block the nerve conduction through the spinal roots. It means that the action of opioids injected into the perineural sheath may be more central due to diffusion or the axonal transport into epidural and subarachnoid spaces. Therefore, opioid transport is optimum for blockades adjacent to the spinal cord. In some studies, injection near the dorsal-root ganglion resulted in very effective impacts.

Chen-Hwan Cherng et al (11) and Cherng CH (12) have shown that epidural fentanyl accelerates the onset time of sensory and motor blockades during the epidural ropivacaine and lidocaine anesthesia. They suggest that fentanyl might stop the nerve conduction in spinal roots.

Kohki Nishikawa and colleagues (5) have confirmed that the onset time of analgesia was prolonged by addition of fentanyl to axiliary brachial plexus blockade. Their study implied that the IV administration of fentanyl has no effects on the rate of success, onset time, or duration of the blockade. On the other hand, the approach to brachial plexus block in this investigation, are axillary block that are more peripheral approach than interscalene block. Thus the conflicting finding in previous studies may be attributed to the site of administration. In this study, similar to Sindjelic's or Enin's study, the centripetal spreading of fentanyl into the adjacent neuroaxial space is possible (13, 14).

Kardash et al (6) have shown that addition of 75 micrograms of fentanyl to mepivacaine supraclavicular blockades has no significant effect on the blockade characteristics.

Animal studies have highlighted the role of inflammation in the expression of peripheral antinociceptive opioid receptors. In these studies, opioid effects can not readily be detected in a normal tissue; however, after initiation of an injury and the emergence of an inflammatory reaction, the preexisting opioid receptors on peripheral nerve terminal and immune cells began to appear. It probably modulates the proliferation of immune cells and their functions (15).

From the clinical aspect, the antinociceptive effects of opioids in some modes of peripheral administration such as intraarticular administration after knee operation or opioid infiltration at the wound site might emerge due to the reduction of the afferent sensory-nerve activity at peripheral nerve terminals by immune-derived opioid peptides. (16, 17).

Perineural blockades such as axiliary blocks, showed equivocal results. In these blockades the axonal opioid receptors are not integrated into the neural membrane and, in noninflammed tissues, the perineurium is intact and opioids cannot easily reach the opioid receptors (18). Therefore in such studies the peripheral opioids'effect is not usually significant (19).

Frazier et al have shown that morphine depressed both sodium and potassium currents associated with the action potential in squid giant axons. Local anesthetics inhibit Na channels current and block the neural transmition. Therefore, combination of local anaesthetics and opioids may effectively inhibit multiple areas of neuronal excitability (20).

In this study the spinal fluid and blood concentrations of fentanyl were not measured. It seems that it is advantageous to measure onset time of block objectively, with computer–assisted infrared thermography camera. Further work is required to define the role of peripherally acting opioids in this situation.

## CONCLUSION

From the results of this study, it appears that addition of 75 µg fentanyl to 1.5% lidocaine solution shorten the onset times of sensory and motor blocks during the interscalene brachial plexus block without any increase in side effects.
